# Molecular identification, phylogeny and geographic distribution of Brazilian mangrove oysters (*Crassostrea*)

**DOI:** 10.1590/S1415-47572010000300030

**Published:** 2010-09-01

**Authors:** Aline Grasielle Costa de Melo, Eduardo Sousa Varela, Colin Robert Beasley, Horacio Schneider, Iracilda Sampaio, Patrick Michael Gaffney, Kimberly S. Reece, Claudia Helena Tagliaro

**Affiliations:** 1 Laboratório de Conservação e Biologia Evolutiva, Instituto de Estudos Costeiros, Campus de Bragança, Universidade Federal do Pará, Bragança, PABrazil; 2 Laboratório de Moluscos, Instituto de Estudos Costeiros, Campus de Bragança, Universidade Federal do Pará, Bragança, PABrazil; 3 Laboratório de Genética e Biologia Molecular, Instituto de Estudos Costeiros, Campus de Bragança, Universidade Federal do Pará, Bragança, PABrazil; 4 College of Marine and Earth Studies, University of Delaware Lewes, DEUSA; 5 Virginia Institute of Marine Science, School of Marine Science, The College of William and Mary, Gloucester Point, VAUSA

**Keywords:** genetic identification, oysters, biogeography, COI gene, Ostreidae

## Abstract

Oysters (Ostreidae) manifest a high degree of phenotypic plasticity, whereby morphology is of limited value for species identification and taxonomy. By using molecular data, the aim was to genetically characterize the species of *Crassostrea* occurring along the Brazilian coast, and phylogenetically relate these to other *Crassostrea* from different parts of the world. Sequencing of the partial cytochrome oxidase c subunit I gene (COI), revealed a total of three species of *Crassostrea* at 16 locations along the Brazilian coast. *C. gasar* was found from Curuçá (Pará state) to Santos (São Paulo state), and *C. rhizophorae* from Fortim (Ceará state) to Florianópolis (Santa Catarina state), although small individuals of the latter species were also found at Ajuruteua beach (municipality of Bragança, Pará state). An unidentified *Crassostrea* species was found only on Canela Island, Bragança. *Crassostrea gasar* and *C. rhizophorae* grouped with *C. virginica,* thereby forming a monophyletic Atlantic group, whereas *Crassostrea* sp. from Canela Island was shown to be more similar to Indo-Pacific oysters, and either arrived in the Atlantic Ocean before the convergence of the Isthmus of Panama or was accidentally brought to Brazil by ship.

## Introduction

Morphological identification of cupped oysters *Crassostrea* to the species level is difficult, due to the intense environmental influence on shell development (Lam and Morton, 2003). Gunter (1950) concluded that, as to the shape of the shell, the oyster is one of the most variable bivalves in the world. Thus, the number of native cupped oyster species from the South American coast remains uncertain. *Crassostrea brasiliana* (Lamarck, 1819) and *Crassostrea rhizophorae* (Guilding, 1828) were initially described from South American Atlantic mangroves (Nascimento, 1991). Great differences in growth rates and larval morphology also lend support to the classification of Brazilian oysters into these two species (Absher, T M. PhD Thesis. Instituto Oceanográfico, USP, 1989). On the other hand, Singarajah (1980) proposed that *C. brasiliana* and *C. rhizophorae* are synonymous, and so, based on morphological and physiological characteristics, described a new species, *Crassostrea paraibanensis,* from the Paraíba river estuary (Paraíba state, Brazil). Furthermore, he suggested the existence of another, as yet, unidentified *Crassostrea* species from the Tijuca lagoon (Rio de Janeiro). Rios (1994) considered all the Brazilian *Crassostrea* morphotypes synonymous with *Crassostrea rhizophorae.*

The only species that was deliberately introduced into Brazilian waters is the Pacific or Japanese oyster, *Crassostrea gigas*, which is cultivated in the cooler, southern waters (Littlepage and Poli, 1999). However, there have been no reports of attempts to cultivate Indo-Pacific oysters in northern Brazil. Along the northern coast (Pará state), some oyster farms have used native oyster larvae from different parts of northeastern Brazil for ongrowing (personal observation).

Biochemical and molecular genetic evidence (Ignacio *et al.*, 2000; Melo *et al.*, 2010) support the existence of two native species of *Crassostrea*, identified as *C. brasiliana* and *C. rhizophorae.* On the other hand, Lapègue *et al.* (2002), on using both molecular evidence (rRNA 16S sequences and RFLP haplotypes) and karyological analysis, discovered the presence of two species from the South American coast, namely *Crassostrea gasar* (Adanson, 1757) and *C. rhizophorae*. Varela *et al.* (2007), based on 16S sequences, agreed with the latter authors and mentioned the presence of a third *Crassostrea* species in north Brazil, that was more closely related to Indo-Pacific oysters.

The inconsistency concerning *C. gasar* and *C. brasiliana* was clarified when a *C. brasiliana* rRNA 16S sequence deposited in the GenBank (DQ839413) by Pie *et al.* (2006) was compared with that of *C. gasar* (AJ312937) studied by Lapègue *et al.* (2002). Both sequences are identical, an indication that they belong to the same species. Furthermore, Nascimento (1991) mentions the presence of *C. brasiliana* in Cananéia (São Paulo state, Brazil) and Ignacio *et al.* (2000) sampled *C. brasiliana* from Paranaguá Bay (Paraná state, Brazil). Moreover, Lapègue *et al.* (2002) obtained samples of *C. gasar* at these same localities. Thus, to date molecular and biochemical evidence confirms the presence of two common species along the Brazilian coast: *C. rhizophorae* and *C. gasar* (*C. brasiliana)*. Based on this evidence, we will refer to *C. brasiliana* by its former name, *C. gasar*.

Molecular and biochemical research, aimed at characterizing oyster species, has intensified worldwide. Ó Foighil *et al.* (1998) and Boudry *et al.* (1998) showed that *Crassostrea angulata*, derived from an Asian population of oysters, had only recently been introduced into Europe. Huvet *et al.* (2000) report evidence of the presence of two stocks of introduced Asian oysters in Europe: *Crassostrea gigas* and *Crassostrea angulata.* Nevertheless, Reece *et al.* (2008) were unable to distinguish between the two, when using COI parsimony analysis. Based on allozyme data, Day *et al.* (2000), besides noting a high degree of homogeneity among cultured populations of *Crassostrea* from Thailand, found that almost all the *Crassostrea belcheri* cultures examined had been contaminated with *Crassostrea lugubris* (= *Crassostrea iredalei*). The 16S rRNA and COI sequences of *Crassostrea* from the Pearl River delta, Hong Kong, were found to be distinct from those of other *Crassostrea* (Lam and Morton, 2003; Boudry *et al.*, 2003), whereat, by using morphological and molecular data, a new species, *C. hongkongensis,* was described (Lam and Morton, 2003).

*Crassostrea* species and their distribution in Brazil are poorly known. Based on COI sequences, our aim was to obtain molecular identification of these very species from the Brazilian coast. According to Hebert *et al.* (2003), from the “Consortium for the Barcode of Life” (Ratnasingham and Hebert, 2007), divergence in COI sequences consistently facilitates the discrimination of closely allied species in all animal phyla, except the Cnidaria. The use of molecular identification of oyster stocks should facilitate monitoring the distribution of native and exotic species, both in the wild and in culture.

## Materials and Methods

Samples of *Crassostrea gasar* and *C. rhizophorae*, previously identified by means of rRNA 16S DNA sequences (Varela *et al.*, 2007), were compared to those published by Lapègue *et al.* (2002) in the GenBank (AJ312937 and AJ312938, respectively), and to the sequence of *C. brasiliana* (= *C. gasar*) deposited in the GenBank (DQ839413) by Pie *et al.* (2006). In order to verify the existence of *Crassostrea paraibanensis*, the rRNA 16S gene of five individuals from the Paraíba river estuary (Paraíba state, Brazil) were sequenced. The results showed that these sequences were identical to those of *C. rhizophorae* published by Varela *et al.* (2007). Therefore, COI genes of oysters from the Paraíba river estuary were not sequenced.

**Figure 1:**
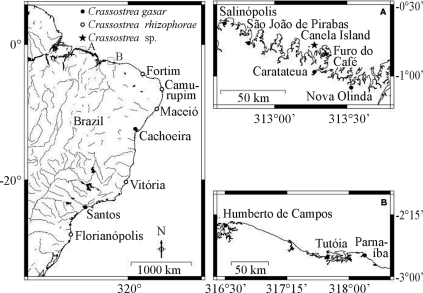
Map of Brazil indicating the sampling locations for mangrove oysters.

The samples of *C. gasar* (n = 215) and *C. rhizophorae* (n = 67) included in our analyses were collected from nine and five localities, respectively (Table 1, Figure 1). Oysters were also collected at Vila Lauro Sodré (00°51'11.2” S, 47°53'24.7” W; municipality of Curuçá, Pará state). Preliminary studies (rRNA16S) revealed these to be *C. gasar* (unpublished data). Young specimens (n = 10; < 2 months old) of a third unidentified *Crassostrea* sp. (n = 10), were obtained from plastic spat collectors at two sites on Canela Island, in the municipality of Bragança (00°47'02” S, 46°43'32.9” W), as well as from a wooden bridge on the Furo do Café tidal channel (00°50'43” S, 46°38'50” W). The scientific names and GenBank sequence accession numbers of oysters compared in the present study are described in Tables 1  and 2. *Crassostrea* sp. collected from Bragança is referred to as *Crassostrea* sp. Canela, the first site where this oyster was found by the authors.

DNA was extracted from the adductor muscle, according to the protocol of Sambrook *et al.* (1989). *Crassostrea rhizophorae* COI sequences were obtained by direct sequencing of PCR amplified fragments, using the primers described by Folmer *et al.* (1994). Samples of *Crassostrea gasar* and *Crassostrea* sp. Canela could only be amplified with a pair of primers designed by C. H. Tagliaro (LCOC-CG-1490 5'- TGTCAACAAATCATT TAGACATTGG-3' and HCOC-CG-2190 5'- TACTTGA CCAAAAACATAAGACATGA-3'), based on the mitochondrial genome sequence of *Crassostrea gigas* (GenBank: NC_001276). The reaction protocol for the samples consisted of initial denaturing at 95 °C for 3 min; 35 cycles of 1 min at 95 °C, 1 min at 45 °C (*C. gasar*), 45.5 °C (*C. rhizophorae*) or 41.4 °C (*Crassostrea* sp. Canela), and 90 s at 72 °C, followed by a final extension at 72 °C for 7 min. The PCR products were purified using ExoSAP-IT® (Pharmacia). DNA sequences were obtained on both strands using dye terminator cycle sequencing reactions (ABI Prism Dye Terminator Cycle Sequencing Ready Reaction, Applied Biosystems), that were subsequently loaded onto an automatic sequencer (Applied Biosystems model 377), according to manufacturer's protocols.

Sequence alignment was carried out with the BioEdit 7 (Hall, 1999) and Clustal X 1.82 (Thompson *et al.*, 1997) programs. Only distinct COI sequences of *C. gasar*, *C. rhizophorae* and *Crassostrea* sp. Canela were aligned with sequences of the other species obtained from the GenBank. Nucleotide frequencies and transition/transversion ratios were obtained by means of Mega 4.0.2 software (Tamura *et al.*, 2007). A saturation test was carried out with the DAMBE 4.2.13 program (Xia and Xie, 2001). A set of aligned sequences is considered to be phylogenetically informative if the observed substitution saturation index (*Iss*) is significantly lower than the critical value of *Iss* (Xia and Xie, 2001). Phylogenetic analyses were undertaken with PAUP* 4.0b10 (Swofford, 2002), using neighbor-joining (NJ) and maximum parsimony (MP), and with PHYML version 3 (Guindon and Gascuel, 2003) using maximum likelihood (ML) methods. MODELTEST 3.07 (Posada and Crandall, 1998) was used for choosing the best model for use in NJ and ML analyses through Hierarchical Likelihood Ratio Tests (HLRTs). Heuristic search was applied in MP and NJ analyses. The robustness of phylogenetic hypotheses obtained, were tested by bootstrapping (Felsenstein, 1985) with 1000 pseudo-replicates for ML and 2000 for NJ and MP. The criterion adopted to evaluate robustness was to consider bootstrap values equal or superior to 90% as being informative.

Two sets of sequence alignments were carried out. For the first set (Analysis I), use was made of only one partial COI sequence of *C. rhizophorae*, one of *C. gasar*, two of *Crassostrea* sp. Canela, and sequences collected from the GenBank: 10 different species of *Crassostrea*, five of *Ostrea*, one of *Ostreola* and one of *Saccostrea*, besides two Lophinae as outgroups (Table 2). For the second set (Analysis II), use was made of 52 different sequences of COI from three Brazilian species of oysters (Table 1), and sequences collected from the GenBank: 10 species of *Crassostrea*, one sequence of *Saccostrea cucullata* (AY038076), with sequences of *Ostrea chilensis* (AF112286) and *Ostrea edulis* (AF120651) as outgroups (Table 2). Analysis I was undertaken to verify whether *Crassostrea* was a monophyletic group, and Analysis II to avoid any influence from saturation that might be caused by Lophinae sequences in the analysis of *Crassostrea* species, as well as to reduce the number of sequences in the analysis itself.

## Results

The divergence in COI sequences was compatible with the presence of three different species of *Crassostrea* along the Brazilian coast. *C. gasar* ( *= C. brasiliana*) was widespread and, in the present study, was found and genetically identified from Vila Lauro Sodré (00°51'11.2” S, 47°53'24.7” W; in the municipality of Curuçá, Pará state) to Santos (São Paulo state). *C. rhizophorae* was found from Fortim to Florianópolis, although some small specimens (< 3 cm) were also encountered and genetically identified from Ajuruteua beach (00°50' S, 46°36' W), in the municipality of Bragança. A third species, *Crassostrea* sp. Canela, which could not be identified by molecular GenBank data comparison, was found only in the municipality of Bragança (Table 1).

**Figure 2:**
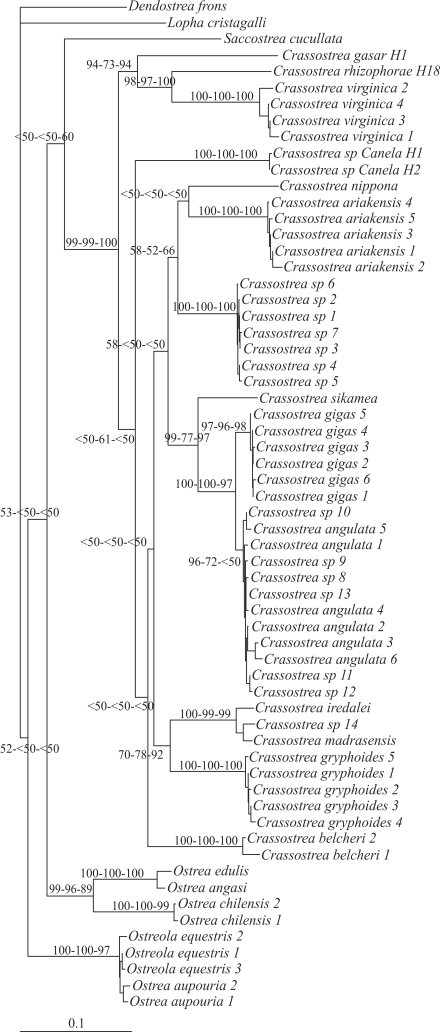
Neighbor-joining (NJ) tree for Ostreinae based on COI*. Dendostrea frons* and *Lophacristagalli* were used as outgroups. Numbers above or below the branches are the bootstrap support values for NJ, Maximum Parsimony and Maximum Likelihood analyses, respectively.

The final alignment of Analysis I sequences was composed of 567 sites (corresponding to nucleotides 169 to 735 of NC007175). Only slight saturation (Iss = 0.236, Iss_c_ = 0.790; p < 0.0001) was detected by saturation testing using COI sequences. The maximum likelihood best fit model for the 62 samples was the General Time-Reversible model – GTR (Lanave *et al.*, 1984; Rodriguez *et al.*, 1990). The settings for the best fit model selected were: base sequences (A = 0.2555, C = 0.1523, G = 0.1860, T = 0.4062); gamma distribution shape parameter (α = 0.6664); substitution model rate matrix (Rmat; A-C = 1.2887, A-G = 13.3881, A-T = 1.1207, C-G = 2.1181, C-T = 18.3779, G-T = 1.0000); and proportion of invariable sites (Pinvar = 0.4527). Forty-eight most parsimonious trees were obtained (best tree score = 1176; CI = 0.369; RI = 0.790). Phylogenetic trees based on ML, NJ (Figure 2) and MP, using COI sequences, strongly supported monophyly of the *Crassostrea* genus (bootstrap values: NJ = 99%, MP = 99%, ML = 100%). The *Crassostrea* sp. Canela specimens were grouped inside the *Crassostrea* clade, although without joining the Atlantic group.

**Figure 3:**
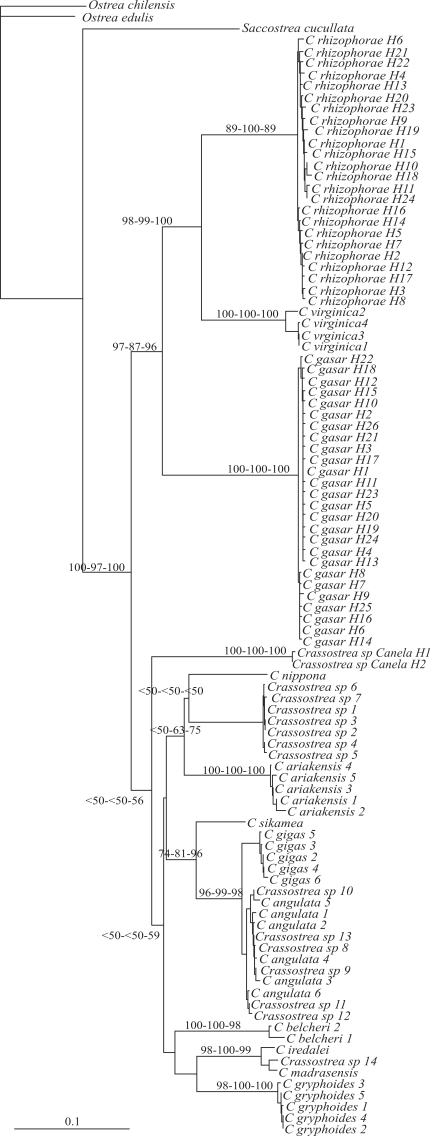
Neighbor-joining (NJ) tree for ten *Crassostrea* species and one *Saccostrea cucullata* based on COI*. Ostrea chilensis* and *Ostrea edulis* were used as outgroups. Numbers above the branches are the bootstrap support values for NJ, Maximum Parsimony and Maximum Likelihood analyses, respectively.

In Analysis II, 538 bp (corresponding to nucleotides 181 to 718 of NC007175) were considered. Intraspecific comparison showed *C. gasar* had 26 different COI sequences (H1 to H26), *C. rhizophorae* 24 (H1 to H24) and *Crassostrea* sp. Canela two (H1 and H2). The aligned *Crassostrea* sequences revealed 226 variable sites, of which 211 were parsimony informative. There were no indels. The average nucleotide base frequencies for *Crassostrea* sequences (n = 97) were 0.376 (T), 0.189 (C), 0.230 (A) and 0.205 (G), whereas the average transition/transversion rate was 1.49. Little saturation of nucleotide sequences (Iss = 0.226, Iss_c_ = 0.798; p < 0.0001) was detected by saturation testing using COI sequences. The maximum likelihood best fit model for the 100 samples was the General Time-Reversible model – GTR (Lanave *et al.*, 1984; Rodriguez *et al.*, 1990). The settings for the best fit model selected were: base sequences (A = 0.2585, C = 0.1453, G = 0.1915, T = 0.4047); gamma distribution shape parameter (α = 0.7680); substitution model rate matrix (Rmat; A-C = 1.2253, A-G = 14.1526, A-T = 0.9369, C-G = 2.5470, C-T = 16.6255, G-T = 1.0000); and proportion of invariable sites (Pinvar = 0.4752). Divergence matrix values in Atlantic *Crassostrea* oyster species ranged from 0.167 (*C. virginica* x *C. rhizophorae*) to 0.261 (*C. gasar* x *C. rhizophorae*), and in Indo-Pacific oysters from 0.021 (*C. angulata* x *C. gigas*) to 0.219 (*C. gryphoides* x *C. angulata*). Molecular data revealed *Crassostrea* sp. Canela to be more similar to Indo-Pacific oysters (d = 0.206 to 0.244) than to Atlantic (d = 0.276 to 0.309). The most similar *Crassostrea* species to *Crassostrea* sp. Canela was *C. belcheri* (d = 0.206 to 0.223) and the least similar *C. gasar* (d = 0.291 to 0.309). One hundred most-parsimonious-trees were obtained (best tree score = 976; CI = 0.406; RI = 0.908). The three Atlantic *Crassostrea* species (*C. gasar, C. virginica* and *C. rhizophorae*) were grouped together with strong support from NJ (Figure 3) and ML (NJ = 97%, MP = 87%, ML = 96%). *C. virginica* and *C. rhizophorae* were clustered (NJ = 98%, MP = 99%, ML = 100%) with *C. gasar* basal. The *Crassostrea* sp. Canela specimens did not group with the Atlantic cupped oysters. In the present study, bootstrap analysis based on COI sequences gave no support to monophyly of the Indo-Pacific species.

## Discussion

As with Varela *et al.* (2007), we also identified the presence of only three species of *Crassostrea* along the Brazilian coast, *C. gasar* (= *C. brasiliana*) and *C. rhizophorae*, both with relatively wide distributions, and *Crassostrea* sp. Canela, found only at two locations in the Bragança region. Although *C. gasar* was not found through our sampling at two different locations in Santa Catarina state, Melo *et al.* (2010) found and genetically identified (16S and ITS-2) *C. brasiliana* (= *C. gasar*) at Florianópolis Island and, more southerly, in the municipality of Laguna (28°30' S; 48°40' W). *C. gasar* was also found and genetically identified (16S) from Paranaguá Bay (Paraná state) by Lapègue *et al.* (2002). *Crassostrea paraibanensis,* described by Singarajah (1980), was not encountered in the Paraíba river estuary, as the samples sequenced from this locality were all molecularly identified as *C. rhizophorae.*

As the morphological identification of *Crassostrea* species is difficult and strongly influenced by the environment (Lam and Morton, 2003), and as molecular analyses suggest the presence of an Indo-Pacific oyster in Pará state and also that *C. gasar* and *C. brasiliana* are synonymous, the urgent need arises for morphological studies to either identify or describe this exotic species, and compare the latter two.

*Crassostrea rhizophorae* has been described from the Caribbean to Uruguay (Rios, 1994). In a recent survey (unpublished data), no *Crassostrea* specimens were encountered from Oiapoque to Macapá (Amapá state). In Brazil, Varela *et al.* (2007) only came across this oyster from Fortim to Florianópolis, without having access to samples further south. In Pará state, some small specimens (unpublished data) were found at Ajuruteua beach, Bragança. Larvae may have arrived by way of ocean currents and afterwards settled, although post-settlement survival may be brief, possibly through *C. rhizophorae* being poorly adapted to highly variable salinity and warm waters. According to Nascimento (1991), this oyster is adapted to living in salinities between 0 and 40 ppt (the optimum between 7.2 and 28.8 ppt). Furthermore, salinities below 18 ppt are apparently deleterious to gonadal and larval development. The Amazonian coast receives an enormous discharge of fresh water (Ekau and Knoppers, 1999), especially during the rainy season, with the consequential reduction in salinity and increase in suspended sediment and turbidity (Müller-Karger *et al.*, 1988). At Ajuruteua beach, Bragança, salinity ranged from 10.9 to 40 ppt between January and December, 2003 (Santos-Filho *et al.*, 2008). On the other hand, Lemos *et al.* (1994) showed that the survival of *C. rhizophorae* veligers is primarily determined by temperature, as shown by 100% mortality at 30 °C during their experiment. In 1997, the temperature of the water in the Caeté Bay, Bragança, which usually ranges from 27 °C to 29 °C, presented a minimum of 23 °C in April (wet season) and a maximum of 31 °C in December (dry season) (Camargo and Isaac, 2005).

Intraspecific comparison indicated a large number of different COI sequences for both *C. rhizophorae and C. gasar. Crassostrea* sp. Canela was shown to have at least two different haplotypes, although there may be more, since the number of specimens was low. The topology of our trees showed that *C. rhizophorae, C. virginica*, and *C. gasar* were closely related, thus in agreement with the trees generated by Lapègue *et al.* (2002), Boudry *et al.* (2003), Lam and Morton (2003) and Varela *et al.* (2007), based on the 16S rRNA gene. On the other hand, our results do not strongly support the monophyly of Indo-Pacific oysters, as previously reported by Lam and Morton (2003), Varela *et al.* (2007), and Reece *et al.* (2008), the latter based on COI sequences.

Molecular sequences of *Crassostrea* sp. Canela were very different in comparison with those of native Brazilian cupped oysters (*C. gasar* and *C. rhizophorae*), and were significantly so in comparison with those of other species of *Crassostrea* deposited in the GenBank (Table 1). *Crassostrea* sp. Canela is more similar to Indo-Pacific oysters, particularly *C. belcheri*, a native of Southeast Asia (including the Philippines, Vietnam, Malaysia and Indonesia), and those of India (Carriker and Gaffney, 1996). It is not known in what manner *Crassostrea* sp. Canela arrived at the mangrove coast of Bragança. It may even have been established in the Atlantic Ocean before the emergence of the Isthmus of Panama. On the other hand, an accidental anthropogenic introduction may have occurred during the colonial period (16^th^ to 19^th^ centuries), when ships belonging to the Portuguese Empire may have brought oysters from the Indo-Pacific to Brazil. The introduction may even have been more recent, via international shipping traffic traveling along the Pará coast towards Manaus, an important industrial and tourist center. Accidental introduction of exotic species has already occurred through either the release of ballast seawater or via external fouling and boring communities on ships (Carlton, 1996). Ó Foighil *et al.* (1998) suggested a similar explanation for the introduction of *C. angulata* to the European coast.

Mechanisms for the introduction of non-indigenous marine organisms were reviewed by Carlton (1989, 1992). Transport by shipping is the most important human activity, culminating in the introduction of exotic organisms, as fouling and boring species, inside the vessels themselves or in ballast water (Carlton, 1992). Moreover, oyster culture has been cited as one of the outstanding agents of exotic species conveyance, through intercontinental transport of species, as larvae or recently settled juveniles (Carlton, 1989; Eldredge, 1994). Many invaders may drastically affect the abundance of species in the recipient community, and in so doing, modify the agents of selection on these species (Vermeij, 1996). Although the introduction of exotic species is on the increase, mainly due to transport via ship ballast water and sediment (Carlton, 1992), there appears to be a lack of adequate legislation governing introductions in most countries. Such a lack of policy may impose ecological risks from the introduction of non-native species for aquaculture (Naylor *et al.*, 2001). Thus, the genetic monitoring of exotic species should be included in the range of measures for use in controlling introductions.

## Figures and Tables

**Table 1:** Species, sample size (n), number of COI haplotypes (H), sampling location and GenBank accession number of Brazilian oysters used in the present study.

Species	n	H	Municipality (Locality)	GenBank accession number
*Crassostrea gasar*	25	7	Salinópolis	HM003499, HM003501, HM003504, HM003507, HM003509, HM003512, HM003513
	32	7	São João de Pirabas	HM003499, HM003502, HM003503, HM003504, HM003505, HM003506, HM003507
	30	5	Bragança (Caratateua)	HM003499, HM003504, HM003508, HM003509, HM003510
	30	3	Augusto Corrêa (Nova Olinda)	HM003499, HM003500, HM003501
	11	4	Humberto Campos	HM003499, HM003517, HM003518, HM003519
	15	4	Tutóia	HM003499, HM003507, HM003515, HM003516
	20	7	Parnaíba	HM003499, HM003516, HM003520, HM003521, HM003522, HM003523, HM003524
	30	4	Cachoeira	HM003499, HM003504, HM003507, HM003511
	22	2	Santos	HM003504, HM003514
*Crassostrea rhizophorae*	9	6	Fortim	HM003475, HM003476, HM003484, HM003490, HM003491, HM003492
	17	8	Camurupim	HM003475, HM003476, HM003493, HM003494, HM003495, HM003496, HM003497, HM003498
	16	8	Maceió	HM003475, HM003476, HM003484, HM003485, HM003486, HM003487, HM003488, HM003489
	14	6	Vitória	HM003475, HM003476, HM003480, HM003481, HM003482, HM003483
	11	5	Florianópolis	HM003475, HM003476, HM003477, HM003478, HM003479
*Crassostrea* sp. Canela	7	2	Bragança (Canela Island)	HM003525, HM003526
	3	1	Bragança (Furo do Café)	HM003525

**Table 2:** GenBank accession numbers of COI sequences of species of Ostreinae and Lophinae used in the analyses.

Subfamily	Species	GenBank accession numbers
Ostreinae	*Crassostrea rhizophorae* H18*	HM003492 ^1^
	*Crassostrea gasar* H1*	HM003499 ^1^
	*Crassostrea* sp1 *-* Canela*	HM003525
	*Crassostrea* sp2 *-* Canela*	HM003526
	*Crassostrea angulata* 1*	AF152567 ^2^
	*Crassostrea angulata* 2*	AJ553907 ^2^
	*Crassostrea angulata* 3*	AJ553908 ^2^
	*Crassostrea angulata* 4*	AY397685 ^2^
	*Crassostrea angulata* 5*	AY397686 ^2^
	*Crassostrea angulata* 6*	AY455664 ^2^
	*Crassostreaariakensis* 1*	AF152569 ^3^
	*Crassostreaariakensis* 2*	AF300617 ^3^
	*Crassostreaariakensis* 3*	AY160752 ^3^
	*Crassostreaariakensis* 4*	AY160753 ^3^
	*Crassostreaariakensis* 5*	AY160754 ^3^
	*Crassostreabelcheri* 1*	AY038077 ^3^
	*Crassostreabelcheri* 2*	AY160755 ^3^
	*Crassostrea gigas* 1	AB033687 ^3^
	*Crassostrea gigas* 2*	AF152565 ^3^
	*Crassostrea gigas* 3*	AF280608 ^3^
	*Crassostrea gigas* 4*	AJ553909 ^3^
	*Crassostrea gigas* 5*	AJ553910 ^3^
	*Crassostrea gigas* 6*	AJ553911 ^3^
	Crassostrea iredalei*	AY038078 ^3^
	Crassostrea nippona*	AF300616 ^3^
	Crassostrea sikamea*	AF152568 ^3^
	*Crassostrea* sp 1*	AJ553912 ^4^
	*Crassostrea* sp 2*	AY160746 ^4^
	*Crassostrea* sp 3*	AY160747 ^4^
	*Crassostrea* sp 4*	AY160748 ^4^
	*Crassostrea* sp 5*	AY160749 ^4^
	*Crassostrea* sp 6*	AY160750 ^4^
	*Crassostrea* sp 7*	AY160751 ^4^
	*Crassostrea* sp 8*	AY249023 ^4^
	*Crassostrea* sp 9*	AY249024 ^4^
	*Crassostrea* sp 10*	AY249025 ^4^
	*Crassostrea* sp 11*	AY249027 ^4^
	*Crassostrea* sp 12*	AY249031 ^4^
	*Crassostrea* sp 13*	AY249032 ^4^
	*Crassostrea* sp 14*	AY249033 ^4^
	*Crassostrea virginica* 1*	AF152566 ^1^
	*Crassostrea virginica* 2*	AY376633 ^1^
	*Crassostrea virginica* 3*	AY376634 ^1^
	*Crassostrea virginica* 4*	AY376635 ^1^
	*Crassostrea gryphoides* 1*	EU007489 ^3^
	*Crassostrea gryphoides* 2*	EU007491 ^3^
	*Crassostrea gryphoides* 3*	EU007488 ^3^
	*Crassostrea gryphoides* 4*	EU007487 ^3^
	*Crassostrea gryphoides* 5*	EU007486 ^3^
	Crassostrea madrasensis*	EU007462 ^3^
	*Ostrea angasi*	AF540598
	*Ostrea aupouria* 1	AY376630
	*Ostrea aupouria* 2	AY376632
	*Ostrea chilensis* 1*	AF112286
	*Ostrea chilensis* 2	AF112289
	Ostrea edulis*	AF120651
	*Ostreola equestris* 1	AY376611
	*Ostreola equestris* 2	AY376618
	*Ostreola equestris* 3	AY376626
	Saccostrea cucullata*	AY038076
Lophinae	*Lopha cristagalli*	AB076908
	*Dendostrea frons*	AB084109

^1^Atlantic species; ^2^Indo-Pacific species introduced into the Atlantic; ^3^Indo-Pacific species; ^4^Unidentified species. *Used in Analyses I and II.
